# Influence of variable domain glycosylation on anti-neutrophil cytoplasmic autoantibodies and anti-glomerular basement membrane autoantibodies

**DOI:** 10.1186/1471-2172-13-10

**Published:** 2012-03-09

**Authors:** Peng-Cheng Xu, Shen-Ju Gou, Xiao-Wei Yang, Zhao Cui, Xiao-Yu Jia, Min Chen, Ming-Hui Zhao

**Affiliations:** 1 Renal Division, Department of Medicine, Peking University First Hospital, Institute of Nephrology, Peking University, Key Laboratory of Renal Disease, Ministry of Health of China, Beijing 100034, China; 2 Department of Nephrology, General Hospital of Tianjin Medical University, Tianjin 300052, China

**Keywords:** Glycosylation, Variable region, ANCA, Anti-GBM

## Abstract

**Background:**

The pathophysiological significance of variable region glycosylation of autoantibodies is still unclear. In the current study, the influence of the variable region N-linked oligosaccharides on the reactivity of three autoantibody specificities was investigated with Sambucus nigra agglutinin (SNA), which mainly binds to oligosaccharides with terminal α2, 6-linked sialic acid on the variable region of IgG.

**Methods:**

Twenty-seven patients with serum positive anti-neutrophil cytoplasmic autoantibodies (ANCA) against myeploperoxidase (MPO) or proteinase 3 (PR3), or autoantibodies against glomerular basement membrane (GBM) were included. Total IgG was isolated and separated into non-SNA-binding and SNA-binding fractions with SNA affinity chromatography. Antigen-specific IgG was purified by immunoaffinity chromatography.

**Results:**

At the same concentration of IgG, the antigen binding level of non-SNA-binding IgG was significantly lower than that of SNA-binding IgG for MPO-ANCA (absorbance value at 405 nm, 0.572 ± 0.590 *vs*. 0.962 ± 0.670, P < 0.001) and for PR3-ANCA (0.362 ± 0.530 *vs*. 0.560 ± 0.531, P = 0.003). The antigen binding level of non-SNA-binding IgG was significantly higher than that of SNA-binding IgG for anti-GBM antibodies (1.301 ± 0.594 *vs*. 1.172 ± 0.583, P = 0.044). The level of variable region glycosylation of total IgG was significantly lower than that of affinity-purified MPO-ANCA (1.021 ± 0.201 *vs*. 1.434 ± 0.134, P = 0.004). The level of variable region glycosylation of total IgG was significantly higher than that of affinity-purified anti-GBM antibodies (1.034 ± 0.340 *vs*. 0.734 ± 0.333, P = 0.007). The SNA-binding fraction of MPO-ANCA-containing IgG and PR3-ANCA-containing IgG induced higher levels of neutrophil oxygen radical production than the corresponding non-SNA-binding fractions (P < 0.001 and P = 0.043, respectively). The level of variable region glycosylation of affinity-purified MPO-ANCA was higher in active AAV than the same patients in remission (P = 0.001).

**Conclusion:**

Characteristics of variable region glycosylation of ANCA and anti-GBM antibodies were different from that of total IgG, which might influence the antigen-binding ability of these antibodies. Variable region glycosylation of ANCA might influence the effect of ANCA-induced neutrophils respiratory burst.

## Background

Anti-neutrophil cytoplasmic antibody (ANCA)-associated vasculitides (AAV) comprise granulomatosis with polyangiitis [GPA, previously termed Wegener's granulomatosis (WG)], microscopic polyangiitis (MPA), Churg-Strauss syndrome (CSS) and renal-limited vasculitis (RLV). ANCAs comprise a group of antoantibodies directed against constituents of granules of neutrophils and lysosomes of monocytes [1]. Proteinase-3 (PR3) and myeloperoxidase (MPO) are the two most important target antigens of ANCAs. In about 4-14% of AAV patients, co-existence of ANCA with anti-glomerular basement membrane (GBM) autoantibodies, directed against the noncollagenous (NC1) domain of α3 chain of type IV collagen (α3(IV)NC1) has been reported [2,3].

IgGs are known to vary in the extent of glycosylation at the highly conserved N-glycosylation sites of the fragment crystallizable (Fc) part. Hyposialylation and hypoglycosylation of serum total IgG-Fc has been reported in AAV [4-6]. Such changes could influence the pathogenetic potential of ANCA [7] but seem to have little, if any, effect on the antigen-binding ability of ANCA [8]. Of note, 15-20% of human IgG molecules bear N-linked oligosaccharides in the fragment antigen binding (Fab) part, depending on the type of variable chain amino acids [9-12]. Since there is no conserved N-linked oligosaccharide site in the constant domain, the N-linked oligosaccharide in the IgG-Fab is actually attached in the variable regions of the light (L) and/or heavy (H) chains [13-17]. Unlike glycosylation of IgG-Fc, N-linked oligosaccharides addition to the Fab region might influence the antigen-binding ability of antibodies [18]. Therefore, we hypothesized that there should be some changes of the variable region glycosylation of ANCA and anti-GBM autoantibodies during antibody affinity maturation.

Sambucus nigra agglutinin (SNA) directed against oligosaccharides with terminal α2, 6-linked sialic acid [19] has been shown to bind strongly to Fab glycans but not to Fc glycans of native IgG [20-24]. In contrast to the Fc glycans, the Fab glycans have been found to be fully sialylated [25,26], allowing us to investigate the characteristics of variable region glycosylation of ANCA and anti-GBM autoantibodies with SNA.

## Methods

### Patients and samples

Plasma exchange fluid from 27 consecutive patients who received plasma exchange treatment at initial onset of active disease, in Peking University First Hospital, was collected. All the plasmapheresis samples were from the first plasmapheresis run. Of the patients included, 10 AAV patients (no.1-10) were MPO-ANCA positive, six patients (no. 11-16) were positive for both MPO-ANCA and anti-GBM antibodies, six patients (no. 17-22) were positive for anti-GBM antibodies without ANCA, and five AAV patients (no. 23-27) were positive for PR3-ANCA (Table 1). The diagnosis of AAV was according to the Chapel Hill Consensus Conference criteria [27].

**Table 1:** General data of the patients at presentation

**Patient No**.	Gender	Age (years)	Serum autoantibodies	Organ involvement	BVAS	Titer of serum autoantibodies (lgT)	SNA binding IgG/IgG (%)
1	F	73	Anti-MPO+	K	10	3.20	7.80
2	M	68	Anti-MPO+	K	12	3.81	8.60
3	M	62	Anti-MPO+	K	14	2.60	6.76
4	F	15	Anti-MPO+	K	12	3.81	10.20
5	F	62	Anti-MPO+	K	16	3.51	7.75
6	F	77	Anti-MPO+	K, L	19	3.2	12.00
7	M	53	Anti-MPO+	K, L	20	3.81	12.0
8	M	72	Anti-MPO+	K, L	18	4.11	13.3
9	F	62	Anti-MPO+	K, L, G, N	22	3.81	13.1
10	M	14	Anti-MPO+	K	11	4.11	9.11
11	M	68	Anti-MPO+, anti-GBM+	K, L	13	2.60, 2.90*	7.20
12	F	13	Anti-MPO+, anti-GBM+	K, L, G	15	3.81, 2.60*	11.60
13	M	68	Anti-MPO+, anti-GBM+	K, L, G, J	20	2.60, 2.90*	11.83
14	M	62	Anti-MPO+, anti-GBM+	K, L, ENT	15	2.00, 3.20*	7.22
15	M	58	Anti-MPO+, anti-GBM+	K, ENT	17	2.90, 3.51*	6.98
16	M	70	Anti-MPO+, anti-GBM+	K, J, ENT	14	3.81, 2.90*	6.34
17	M	19	Anti-GBM+	K, L	--	3.81	13.05
18	M	26	Anti-GBM+	K, L	--	3.51	6.68
19	M	24	Anti-GBM+	K, L	--	2.90	22.37
20	M	27	Anti-GBM+	K	--	3.20	8.20
21	M	17	Anti-GBM+	K	--	3.20	6.86
22	M	26	Anti-GBM+	K	--	2.90	12.44
23	M	72	Anti-PR3+	K, L, J, ENT	18	2.00	18.68
24	M	67	Anti-PR3+	K	9	2.00	4.00
25	M	68	Anti-PR3+	K, L, J, ENT	21	2.60	6.30
26	F	21	Anti-PR3+	K, L,	16	2.00	14.7
27	M	50	Anti-PR3+	K, ENT	14	2.90	8.23

**[Abbreviations] **BVAS: Birmingham Vasculitis Activity Score; ENT: ear, nose and throat; G: gastrointestinal tract; J: joint; K: kidney; L: lung or respiratory system; MPO: myeloperoxidase; N: nervous system; PR3: proteinase 3; SNA: Sambucus nigra agglutinin.

*: titer of anti-GBM antibody.

Among the above patients who were MPO-ANCA positive and were diagnosed as AAV, 5 patients (patient no. 1, 3, 4, 8 and 10) still had positive MPO-ANCA in remission. The plasma samples of these 5 patients were collected during remission.

Plasma collected from 20 healthy blood donors served as normal control samples. All plasma samples were collected at presentation and stored at -20°C until use. The research protocol was in compliance with the Declaration of Helsinki and approved by the Ethnics Committee of Peking University First Hospital. Written inform consent was obtained from each participant.

### Detection of ANCA and anti-GBM antibodies

ANCA tests were performed by both indirect immunofluorescence (IIF) assay and antigen-specific enzyme-linked immunosorbent assay (ELISA). Standard IIF assay were performed according to the manufacturer (EUROIMMUN, Lübeck, Germany). In antigen-specific ELISAs, highly purified PR3 and MPO [28] were used as solid phase ligands. Anti-GBM antibodies were detected by ELISA as previously described [29].

### Detection of the variable region glycosylation of plasma total IgG with SNA

In brief, plates were coated with recombinant protein G (Biovision, Mountain View, CA, USA) at 0.087 μg/ml in 0.05 mol/L bicarbonate buffer, pH 9.6, 1 hr at 37°C. Plasma diluted 1: 3600 with 0.01 mol/L phosphate-buffered saline (PBS) containing 0.1% Tween-20 (PBST) was added to the wells in duplicate and incubated at 37°C for 1 hr. Then biotinylated SNA (Vector Laboratories, Burlingame, CA, USA) diluted 1: 2,000 in PBST was added for 1 hr followed by incubation for 1 hr with alkaline phosphatase-conjugated streptavidin (Sigma, St. Louis, MO, USA) diluted 1:2,000 in PBST. The p-nitrophenyl phosphate (pNPP, 1 mg/ml; Sigma) was used in substrate buffer [1 M diethanolamine and 0.5 mM MgCl_2 _(pH 9.8)]. Color development was measured spectrophotometrically at 405 nm (Bio-Rad, Tokyo, Japan). In each step, the volume was 100 μL and the plates were washed three times with PBST between steps. All samples were tested in duplicate.

### Purification of IgG fractions

IgG fractions were purified by protein G affinity column (Amersham Pharmacia, Sweden) with PBS as starting buffer and 0.1 mol/L glycine, pH 2.7 as eluting buffer, at a flow rate of 1 mL/min at room temperature. IgG was eluted and neutralized to pH 7.0 with 2 mol/L Tris-HCl, pH 9.0 immediately, and dialyzed against PBS. To obtain IgG-Fab and IgG-Fc fragments, purified IgG was digested with papain (Sigma) after which the digest was loaded onto protein A affinity column (Amersham Pharmacia, Sweden).

### Purification of autoantibodies with immunoaffinity chromatography

In brief, IgG fractions containing anti-MPO antibodies and anti-GBM antibodies were applied to the affinity column coupled with purified native MPO [28] and α3(IV)NC1 respectively. Antigen-specific fractions were eluted with 0.05 mol/L glycine and 0.5 mol/L NaCl (pH 2.7), neutralized to pH 7.0, concentrated, and dialyzed against PBS.

### Detection of SNA binding to intact IgG, IgG-Fab and IgG-Fc

The assay was performed as described by Dalziel et al [21], with some minor modifications. Intact IgG, prepared Fab and Fc fragments of IgG were loaded on a SDS-polyacrylamide gel (SDS-PAGE) separately. Electrophoresis was performed for 40-60 min in Tris-Glycine buffer. The proteins were transferred onto nitrocellulose (Schleicher & Schuell, Dassel, Germany) and blocked overnight at 4°C in PBS (pH 7.4)/0.2% Tween 20/1.0% bovine serum albumin (PTB solution). The blot was then incubated with biotinylated SNA (Vector, 5 mg/mL in PTB solution), washed three times (15 min each) with PBST, incubated for 1 hr with streptavidin-horseradish peroxidase (Vector, 1: 2000 dilution in PTB solution), washed three times as before, and then detected using enhanced chemiluminescence (ECL) and light sensitive film. To confirm the position of IgG, Fab and Fc fragments, horseradish peroxidase-labeled polyclonal goat antibodies against intact IgG (Sigma), Fab (Sigma) and Fc (Sigma) were used instead of biotinylated SNA after blocking. Then the same detecting procedure was followed.

### Lectin affinity chromatography

The assay was performed as described by Franco et al. [30], with some minor modifications. IgG fractions were applied to the affinity column coupled with SNA (Vector), at a flow rate of 0.3 mL/min, with PBS as starting buffer. The eluted fractions were eluted with 0.5 mol/L lactose in PBS followed by 0.5 mol/L lactose in 0.2 mol/L acetic acid buffer (pH 3.0). The eluted fractions were neutralized to pH 7.0, concentrated, and dialyzed against PBS.

### Detection of the antigen binding levels of non-SNA-binding IgG and SNA-binding IgG

The assay was performed as described above, with some minor modifications. After antigen was coated on plates, the IgG diluted 100 μg/mL with PBST was added. Every sample was tested in two parallel wells. All assays were taken in duplicate.

To further confirm the influence of variable glycosylation on the antigen-binding level of IgG, the SNA-binding IgG samples were digested at 37°C for 48 hr with neuraminidase (Sigma), which could remove the terminal α2, 6-linked sialic acid from the N-glycan of IgG, in reaction buffer, pH 5.5. Alternatively, the proteins were exposed to endoglycosidase F2 (Sigma), which could release the whole N-glycan from the native IgG-Fab [31], at 37°C for 1 hr in reaction buffer, pH 4.5.

### Detection of avidity constant of non-SNA-binding IgG and SNA-binding IgG

The avidity constant (aK) was determined as the reciprocal value of the antigen (MPO or α3(IV)NC1) molar concentration in the liquid phase resulting in 50% inhibition of antigen-antibody binding in solid phase ELISA, as described in our previous study [32]. Briefly, the appropriate IgG dilution which gave an OD value of about 0.7 in the standard ELISA was determined first for each IgG (non-SNA-binding IgG or SNA-binding IgG). The competitive binding assay was performed by incubating the appropriately diluted IgG with increasing amounts of purified antigen (0.1 mg/L-100 mg/L) in PBST for 2 hr at 37°C. The mixture was then transferred to antigen-coated plates for the standard ELISA procedure. All assays were performed in duplicate.

### Detection of variable region glycosylation levels of total IgG and affinity-purified autoantibodies

The assay was performed as described above, with some minor modifications. Total IgG or purified autoantibodies were diluted at 2 μg/mL in 0.05 mol/L bicarbonate buffer, pH 9.6 and coated on plates at 37°C for 1 hr. The following steps were the same as above from the step in which biotinylated SNA was added. Each sample was tested in duplicate.

### Measurement of respiratory burst in neutrophils by oxidation of dihydrorhodamine (DHR) to rhodamine

The generation of reactive oxygen radicals using DHR was assessed as described previously [33], with some minor modification. In brief, neutrophils (2.5 × 10^6^/mL HBSS) were incubated with cytochalasin B (5 μg/mL, Sigma) for 5 min at 37°C to enhance the oxygen radical production. Then, neutrophils were loaded with 0.05 mM DHR (Sigma) and 2 mM sodium azide (NaN_3_) at 37°C and primed with TNFα (2 ng/mL) for 15 min at 37°C. The non-SNA-binding and the SNA-binding fraction of anti-MPO antibodies-containing IgG and anti-PR3 antibodies-containing IgG were added with a final concentration of 100 μg/mL. Monoclonal mouse anti-MPO antibody (5 μg/mL, Abcam, Cambridge, MA, USA) and monoclonal mouse anti-PR3 antibody (5 μg/mL, Abcam) were used as positive controls. Then the reaction was stopped after 60 min by addition of 3 mL of ice-cold HBSS/1%BSA. We analyzed samples using Calibur flow cytometer (BD FACSCalibur). Data were collected from 20,000 cells per sample. The mean fluorescence intensity (MFI), representing the amount of generated oxygen radicals, was reported. All assays were performed in duplicate.

### Statistical analysis

Quantitative data were expressed as mean ± SD and were evaluated using *t*-test or one-way ANOVA analysis as appropriate. The Pearson test was used for correlation analysis. A P-value of less than 0.05 was considered significant. Analysis was performed with SPSS statistical software package (version 11, Chicago, IL, USA).

## Results

### General data of the patients

Demographic and clinical data of the patients included are listed in Table 1. The average titer of anti-MPO antibodies, anti-GBM antibodies and anti-PR3 antibodies was 3.36 ± 0.64, 3.13 ± 0.35 and 2.30 ± 0.42, respectively. The titer of anti-PR3 antibodies was significantly lower than that of anti-MPO antibodies and anti-GBM antibodies (P < 0.001 and P = 0.006, respectively).

### Identification of binding between SNA and IgG-Fab

**Figure 1:**
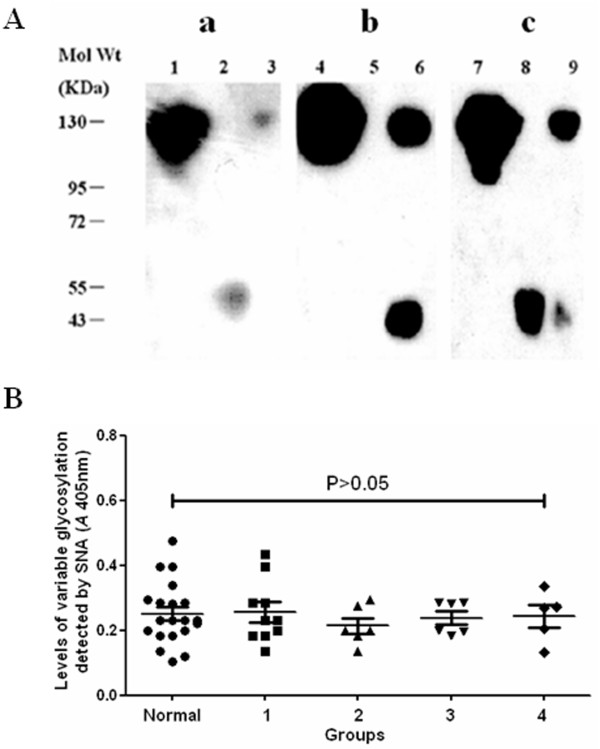
**Identification of the binding specificity of SNA**. A: Western-blot analysis of intact IgG, IgG-Fab and IgG-Fc after papain digestion. a: Detection with mouse polyclonal anti-Fab antibodies. Lane 1: intact IgG; lane 2: flow-through fraction of protein A-Sepharose; lane 3: eluted fraction of protein A-Sepharose. b: Detection with mouse polyclonal anti-Fc antibodies. Lane 4: intact IgG; lane 5: flow-through fraction of protein A-Sepharose; lane 6: eluted fraction of protein A-Sepharose. c: Detection with SNA. Lane 7: intact IgG; lane 8: flow-through fraction of protein A-Sepharose; lane 9: eluted fraction of protein A-Sepharose. B: Comparison of variable region glycosylation levels of plasma total IgG between patients and normal controls. Normal: 20 normal controls; group 1: 10 patients with anti-MPO antibodies; group 2: 6 patients with both anti-GBM antibodies and anti-MPO antibodies; group 3: 6 patients with anti-GBM antibodies without anti-MPO antibodies; group 4: 5 patients with anti-PR3 antibodies.

After incubation with papain, intact IgG was partially digested. The resulting digest was separated into two parts with protein A chromatography, i.e. Fab fragments (flow-through fraction) and Fc fragments together with undigested IgG (eluted fraction). Proper separation was confirmed by Western blot using polyclonal mouse anti-Fab antibodies and polyclonal mouse anti-Fc antibodies (Figure 1). SNA mainly recognized Fab (lane 8) and weakly recognized Fc (lane 9), as has been described previously [20,21].

### Comparison of the variable glycosylation levels of plasma total IgG between patients and normal controls

The variable region glycosylation levels of plasma total IgG of 20 normal controls, 10 patients with anti-MPO antibodies (no. 1-10), 6 patients with both anti-MPO antibodies and anti-GBM antibodies (no.11-16), 6 patients with anti-GBM antibodies (no.17-22), and 5 patients with anti-PR3 antibodies (no. 23-27) was 0.253 ± 0.102, 0.256 ± 0.096, 0.215 ± 0.060, 0.238 ± 0.050 and 0.245 ± 0.088, respectively [expressed by the absorbance value at 405 nm (*A*405)]. There was no significant difference in the variable region glycosylation levels of plasma total IgG among these groups (P > 0.05) (Figure 1B).

### SNA-affinity chromatography

The percentages of the SNA-binding fraction in total IgG for the different groups were as follows: 9.23 ± 5.63% (for 5 healthy blood donors), 10.06 ± 2.39% (for 10 patients with anti-MPO antibodies), 8.53 ± 2.49% (for 6 patients with both anti-GBM antibodies and anti-MPO antibodies), 11.60 ± 5.95% (for 6 patients with anti-GBM antibodies and without anti-MPO antibodies) and 10.38 ± 6.11% (for 5 patients with anti-PR3 antibodies). There was no significant difference in the percentage of the SNA-binding fraction in total IgG among these groups (P = 0.575).

### Comparison of antigen-binding levels between non-SNA-binding IgG and SNA-binding IgG

**Figure 2:**
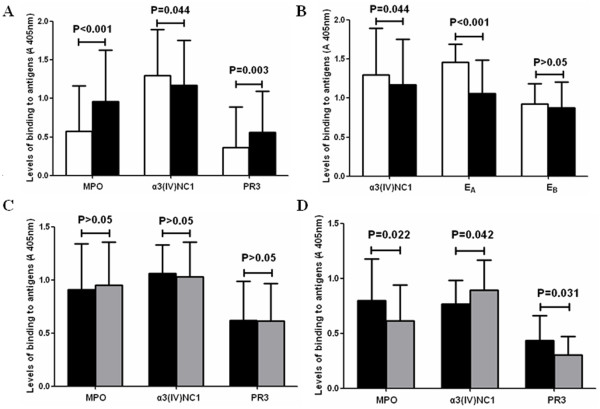
**Comparison of antigen-binding levels between non-SNA-binding IgG and SNA-binding IgG**. A: Comparison of binding levels to MPO, α3(IV)NC1 and PR3 between non-SNA-binding IgG and SNA-binding IgG. Open squares represented non-SNA-binding IgG; Filled squares represented SNA-binding IgG. B: Comparison of binding levels to α3(IV)NC1, E_A _and E_B _between non-SNA-binding IgG and SNA-binding IgG. Open squares represented non-SNA-binding IgG; Filled squares represented SNA-binding IgG. C: Influence of neuraminidase treatment on the antigen-binding levels of SNA-binding IgG. Filled squares: before treatment; gray squares: after treatment D: Influence of endoglycosidase F2 treatment on the antigen-binding levels of SNA-binding IgG. Filled squares: before treatment; gray squares: after treatment.

For anti-MPO antibodies-containing IgG (patients no. 1-16), the binding level to MPO of non-SNA-binding fractions was significantly lower than that of SNA-binding fractions at the same concentration of IgG (0.572 ± 0.590 *vs*. 0.962 ± 0.670, P < 0.001). For anti-PR3 antibodies-containing IgG (patients no. 23-27), the binding level to PR3 of non-SNA-binding fractions was significantly lower than that of SNA-binding fractions at the same concentration of IgG (0.362 ± 0.530 *vs*. 0.560 ± 0.531, P = 0.003). For anti-GBM antibodies-containing IgG (patients no. 10-22), the binding level to α3(IV)NC1 of non-SNA-binding fractions was significantly higher than that of SNA-binding fractions at the same concentration of IgG (1.301 ± 0.594 *vs*. 1.172 ± 0.583, P = 0.044) (Figure 2A).

Since anti-GBM antibodies mainly recognize two regions on α3(IV)NC1 (E_A_, residues 17 to 31 and E_B_, residues 127 to 141) [34], we compared the antigen binding levels to recombinant EA and E_B _[35] between non-SNA-binding IgG and SNA-binding IgG of patients with positive anti-GBM antibodies. As shown in Figure 2B, the binding level to E_A _of non-SNA-binding fractions was significantly higher than that of SNA-binding fractions (1.462 ± 0.230 *vs*. 1.060 ± 0.431, P < 0.001), while the binding level to E_B _of non-SNA-binding fractions was similar to that of SNA-binding fractions (0.923 ± 0.265 *vs*. 0.879 ± 0.325, P > 0.05).

After SNA-binding IgG was treated with neuraminidase, there was no significant change in antigen-binding level (Figure 2C). However, when SNA-binding IgG was treated with endoglycosidase F2, the binding level to MPO of anti-MPO antibodies-containing IgG and the binding level to PR3 of anti-PR3 antibodies-containing IgG decreased (P = 0.022 and P = 0.031, respectively), while the binding level to α3(IV)NC1 of anti-GBM antibodies-containing IgG increased (P = 0.042) (Figure 2D).

### Comparison of the avidity constant between non-SNA-binding IgG and SNA-binding IgG

**Figure 3:**
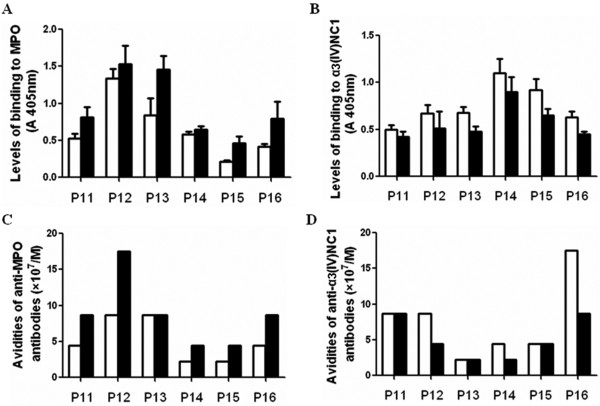
**Comparison of avidity constant of anti-MPO and anti-α3(IV)NC1 antibodies between non-SNA-binding IgG and SNA-binding IgG**. A and B: Comparison of antigen-binding levels of anti-MPO antibodies and anti-α3(IV)NC1 antibodies between non-SNA-binding IgG and SNA-binding IgG. C and D: Comparison of avidity constant of anti-MPO antibodies and anti-α3(IV)NC1 antibodies between non-SNA-binding IgG and SNA-binding IgG. Open squares represent non-SNA-binding IgG; Filled squares represent SNA-binding IgG.

Patients no. 11-16 who had both anti-MPO antibodies and anti-α3(IV)NC1 antibodies were included in this analysis. As shown in Figure 3, the MPO-binding level of non-SNA-binding IgG was lower than that of SNA-binding IgG for all 6 patients, while the MPO-binding avidity constant of non-SNA-binding IgG was lower than that of SNA-binding IgG in 5 out of 6 patients. The α3(IV)NC1-binding level of non-SNA-binding IgG was higher than that of SNA-binding IgG for all 6 patients, while the α3(IV)NC1-binding avidity constant of non-SNA-binding IgG was higher than that of SNA-binding IgG in 3 out of 6 patients.

### Effects of non-SNA-binding ANCA-IgG and SNA-binding ANCA-IgG on respiratory burst induction in neutrophils

**Figure 4:**
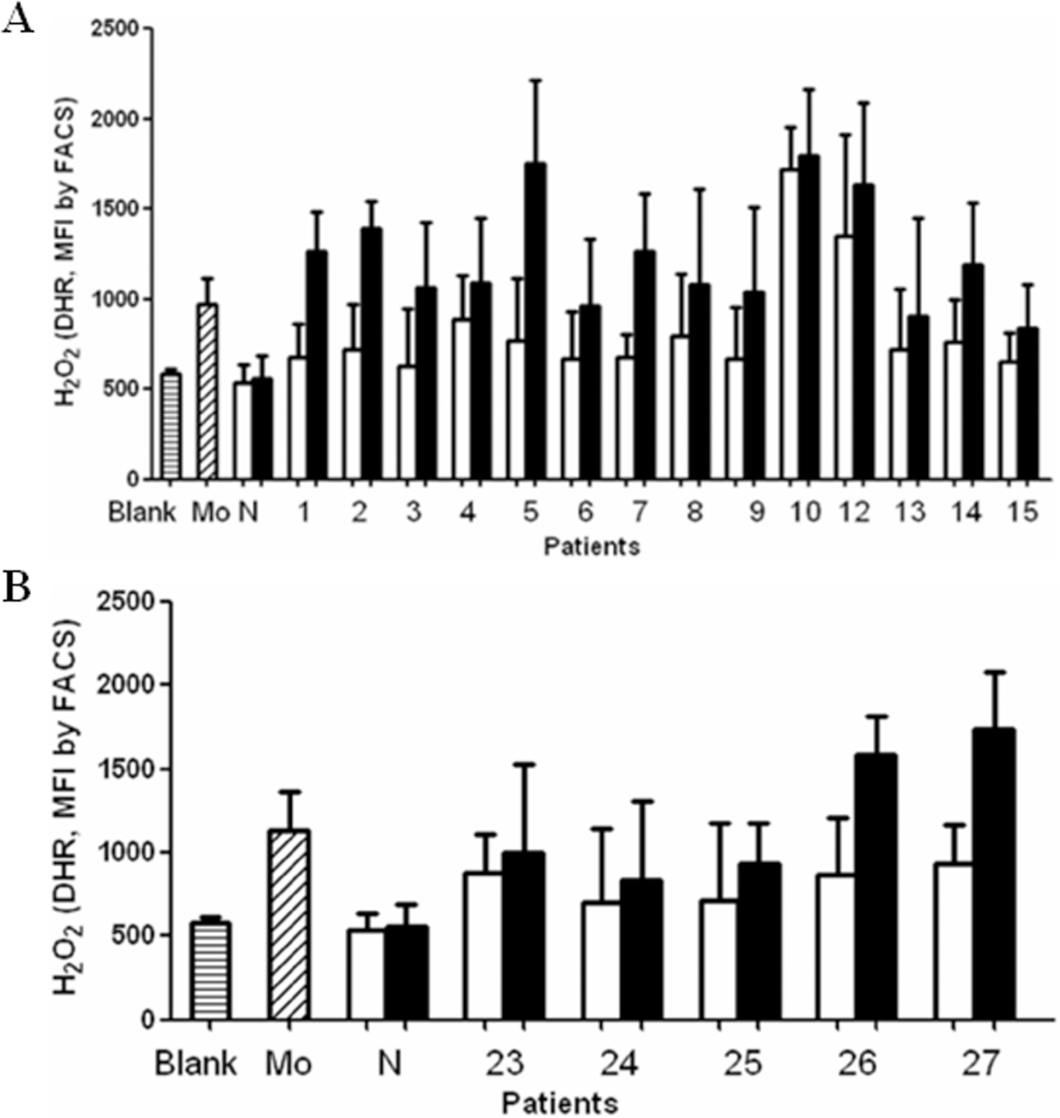
**Comparison of effects on respiratory burst of neutrophils between non-SNA-binding IgG and SNA-binding IgG**. A: Respiratory burst of neutrophils induced by anti-MPO antibodies. Mo: monoclonal mouse anti-MPO antibody. N: normal IgG. Because of limited sample volume, two patients with anti-MPO antibodies and anti-GBM antibodies (patients 11 and 16) were not included in this analysis. Open squares represent non-SNA-binding IgG; Filled squares represent SNA-binding IgG. B: Respiratory burst of neutrophils induced by anti-PR3 antibodies. Mo: monoclonal mouse anti-PR3 antibody. N: normal IgG. Open squares represent non-SNA-binding IgG; Filled squares represent SNA-binding IgG.

Ten patients with anti-MPO antibodies (patient no. 1-10), 4 patients with both anti-MPO antibodies and anti-GBM antibodies (patient no. 12-15) and 5 patients with anti-PR3 antibodies (patient no. 23-27) were enrolled in this analysis. In the absence of IgG, the baseline level of the respiratory burst of neutrophils was 586.50 ± 28.21 (expressed by MFI). Moreover, IgG of a healthy blood donor did not significantly activate oxygen radical production in neutrophils irrespective of the variable region glycosylation status. Both monoclonal mouse anti-MPO antibody and anti-MPO antibodies-containing IgG of patients enhanced the respiratory burst of neutrophils. Compared with the non-SNA-binding fractions of anti-MPO antibodies-containing IgG, the SNA-binding fractions induced a significantly higher level of the respiratory burst of neutrophils (1025.14 ± 322.09 *vs*. 843.00 ± 326.36, P < 0.001) (Figure 4A). Similar results were obtained when comparing the non-SNA-binding fractions of anti-PR3 antibodies-containing IgG and the SNA-binding fractions for respiratory burst induction in neutrophils (1218.60 ± 414.62 *vs*. 817.80 ± 105.14, P = 0.043) (Figure 4B).

### Antigen-specific IgG separation with immunoaffinity chromatography

The percentage of purified anti-MPO antibodies in total IgG from patient no. 1-16 was 1.53 ± 0.66%. The percentage of the purified anti-α3(IV)NC1 antibodies in total IgG from patient no. 11-22 was 1.06 ± 0.55%. There was no significant difference between the two groups (P = 0.838).

### Comparison of levels of variable region glycosylation between total IgG and antigen-specific IgG

**Figure 5:**
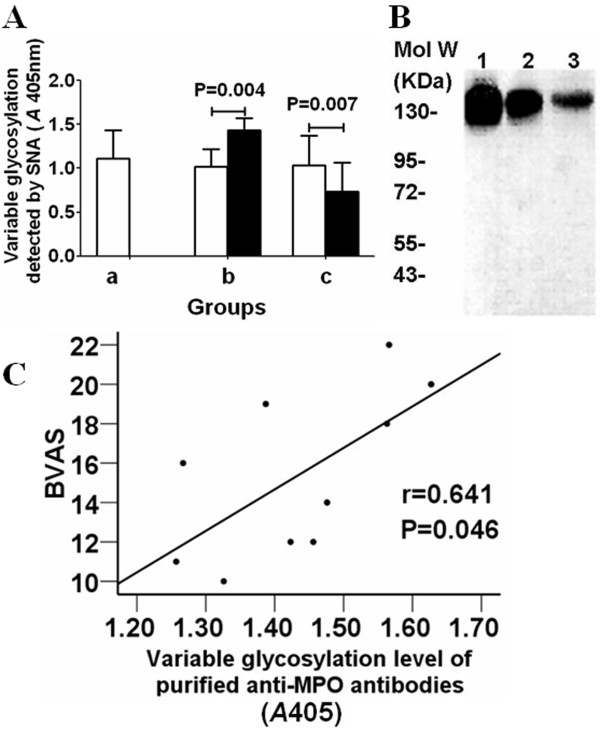
**Comparison of levels of variable glycosylation between total IgG and affinity-purified autoantibodies**. A: Comparison of levels of variable region glycosylation between total IgG and purified autoantibodies in ELISA. Group a: healthy controls. Open squares represent total IgG. Group b: patients with positive anti-MPO antibodies. Open squares represent total IgG; Filled squares represent affinity-purified anti-MPO antibodies. Group c: patients with positive anti-GBM antibodies. Open squares represent total IgG; Filled squares represent affinity-purified anti-α3(IV)NC1 antibodies. B: Comparison of levels of variable glycosylation between total IgG and purified autoantibodies by Western-blot analysis. The concentration of all samples was 1 μg/mL. Lane 1: mixture of affinity-purified anti-MPO antibodies from patient 11-16; lane 2: mixture of total IgG from patient 11-16; lane 3: mixture of affinity-purified anti-α3(IV)NC1 antibodies from patient 11-16. C: Correlation between the levels of variable glycosylation of anti-MPO antibodies and BVAS.

The variable region glycosylation level of total IgG from MPO-ANCA positive patients was comparable to that of total IgG from 5 healthy donors (*A*405, 1.021 ± 0.201 vs. 1.107 ± 0.326, P > 0.05), but was significantly lower than that of affinity-purified anti-MPO antibodies (1.021 ± 0.201 vs. 1.434 ± 0.134, P = 0.004). The variable region glycosylation level of total IgG from anti-GBM antibody positive patients was also comparable to that of total IgG from 5 healthy donors (1.034 ± 0.340 vs. 1.107 ± 0.326, P > 0.05), but was significantly higher than that of affinity-purified anti-GBM antibodies (1.034 ± 0.340 vs. 0.734 ± 0.333, P = 0.007) (Figure 5A).

Then the results were further confirmed with Western blot analysis. Six patients who had both anti-MPO antibodies and anti-GBM antibodies (patient no. 11-16) were included in this analysis. Total IgG, purified anti-MPO antibodies and purified anti-GBM antibodies of these patients were mixed respectively and were analyzed in Western blot analysis. As shown in Figure 5B, the level of variable glycosylation of mixed total IgG was lower than that of mixed purified anti-MPO antibodies, but was higher than that of mixed purified anti-GBM antibodies.

### Correlation between levels of variable glycosylation of anti-MPO antibodies and clinical parameters

Correlation analysis was performed for patients no. 1-10 who were MPO-ANCA positive but negative for anti-GBM antibodies. No correlation between the levels of variable region glycosylation of affinity-purified MPO-ANCA and the titers of MPO-ANCA was found (r = 0.149, P = 0.682). No correlation between the level of variable region glycosylation of affinity-purified MPO-ANCA and the percentage of the SNA-binding IgG in total IgG was found (r = 0.628, P = 0.052). However, the level of variable region glycosylation of affinity-purified MPO-ANCA correlated with Birmingham Vasculitis Activity Score (BVAS) [36] (r = 0.641, P = 0.046) (Figure 5C). The number of PR3-ANCA positive patients was to low for correlation analysis.

### Comparison of variable region glycosylation of anti-MPO antibodies between patients with active AAV and in remission

**Figure 6:**
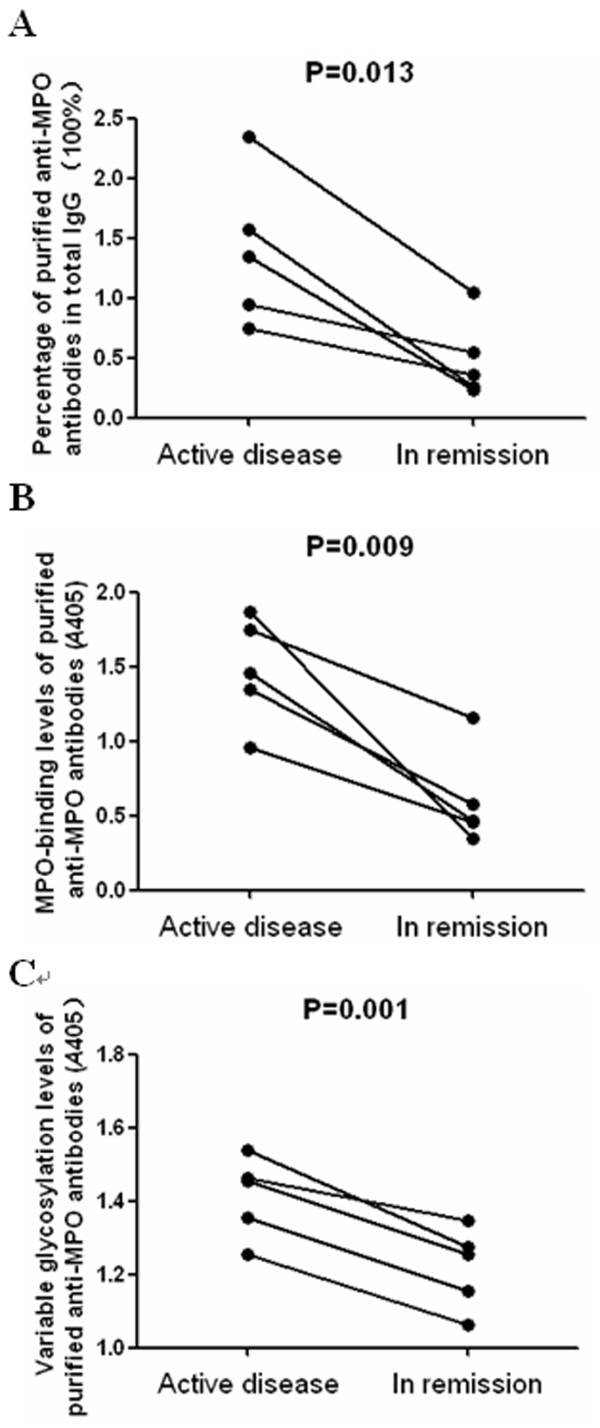
**Comparison of immune characteristics of anti-MPO antibodies between AAV patients in active phase and in remission**. A: Comparison of percentage of affinity-purified anti-MPO antibodies in total IgG between AAV patients in active phase and in remission. B: Comparison of antigen-binding levels of affiniy-purified anti-MPO antibodies between AAV patients in active phase and in remission. C: Comparison of levels of variable region glycosylation of affiniy-purified anti-MPO antibodies between AAV patients in active phase and in remission.

Plasma samples of 5 patients (patient no. 1, 3, 4, 8 and 10) who had positive MPO-ANCA in the remission stage of AAV were obtained.. As shown in Figure 6, the percentage of the purified MPO-ANCA in total IgG was significantly higher in active phase than the same patients in remission (1.386 ± 0.624% vs. 0.487 ± 0.332%, P = 0.013). The antigen-binding level of affinity-purified MPO-ANCA was significantly higher in active phase than the same patients in remission (1.473 ± 0.359 vs. 0.599 ± 0.321, P = 0.009). The level of variable region glycosylation of affinity-purified MPO-ANCA was significantly higher in active phase than the same patients in remission (1.414 ± 0.109 vs. 1.217 ± 0.110, P = 0.001).

## Discussion

Previous studies on the variable region glycosylation of IgG have been performed mainly on monoclonal antibodies from animals or total IgG from healthy individuals [37,38]. To our knowledge, our study is the first investigation on the glycosylation status of the variable region of disease-associated antigen-specific IgGs. Previous studies by Holland et al. [25] have shown that while the oligosaccharides released from ANCA-containing IgG-Fc were hypogalactosylated and hyposialylated, those released from IgG-Fab were normally galactosylated and sialylated. In this study the authors did not report any difference in variable region glycosylation between ANCA-containing IgG and normal IgG upon analysis of total serum-derived IgG. In the current study, when we compared the variable region glycosylation levels of total IgG in plasma between patients and normal individuals, no significant difference was found either. However, affinity-purified anti-MPO antibodies were found to have a significantly higher level of variable region glycosylation than total IgG, while purified anti-GBM antibody was found to have a significantly lower level of variable region glycosylation than total IgG.

In the current study, incubation with neuraminidase did not significantly change the antigen-binding ability of SNA-binding IgG, while incubation with endoglycosidase F2 significantly changed the antigen-binding ability of SNA-binding IgG. It indicated that the variable region glycan molecule, but not the terminal sialic acid, influenced the antigen-binding ability of autoantibodies It also indicated that electrostatic forces are not a major determinant, because the isoelectric point of α3(IV)NC1 (calculated isoelectric point 8.9 [39]) is between the isoelectric points of MPO and PR3 (11.0 and 7.9 for MPO and PR3, respectively [40,41]). How oligosaccharides in the variable region influence antigen-antibody binding is not clear yet. In a previous study, it was proposed that oligosaccharides in the variable region affect the antigen-binding ability by influencing hydrophilic interactions between the antibody and antigen [42]. Amino acid sequences of the variable regions of IgG are highly variable and are influenced by a number of factors. Initially, the rearrangement of VH and VL genes occurs in the bone marrow. Then after B cells encounter antigen, the somatic mutation machinery is activated in the germinal center [43]. Interestingly, sequence analysis has shown that there is no potential N-linked glycosylation site [Asn-X-Ser/Thr (X is any amino acid except Pro, Asp or Glu)] in germline V sequences, suggesting that variable region glycosylation sites mainly arise from somatic mutations [44-46]. Since the N-linked glycosylation site in the variable region is generated during the process of affinity maturation [47], we speculate that the type of antigen is one of the determinants of oligosaccharide distribution in the variable region of the antibody. Moreover, since the oligosaccharide distribution of the variable region can influence the antigen-antibody binding, it could also influence the process of affinity maturation of the antibody. We speculate that if a variable region sequence, with or without potential glycosylation sites, has higher affinity for an antigen, this sequence will most likely be amplified during affinity maturation.

It has been suggested that hyposialylation of the ANCA antibody Fc part increases the ability of ANCA to induce the respiratory burst in neutrophils [7]. In the present study, we showed that ANCA IgG with a high level of variable region glycosylation had a higher avidity constant and an increased ability to induce the respiratory burst in neutrophils. Furthermore, the level of variable glycosylation of purified MPO-ANCA positively correlated with disease activity. Moreover, MPO-ANCA from patients in remission demonstrated a significantly lower level of variable region glycosylation compared to MPO-ANCA from patients with active disease. These results suggest that the level of variable region glycosylation of ANCA might be a potent biomarker reflecting disease activity in AAV.

## Conclusions

In conclusion, the characteristics of variable region glycosylation of ANCA and anti-GBM antibodies were different from that of total IgG, which might influence the antigen-binding ability of these antibodies. Variable region glycosylation of ANCA might influence the effect of ANCA-induced neutrophils respiratory burst.

## Competing interests

The authors declare that they have no competing interests.

## Authors' contributions

**PCX**: ELISA analysis, FACS analysis, experimental design and writing; **SJG**: ELISA analysis, immunoaffinity chromatography, lectin affinity chromatography; **XWY**: helped in the design and in the conduction of the experiments; **ZC**: sample collection; helped in the design and in the conduction of the experiments; **XYJ**: sample collection; **MC and MHZ**: designed and directed the study. All authors read and approved the manuscript.
